# In Vitro Osteoinductivity Assay of Hydroxylapatite Scaffolds, Obtained with Biomorphic Transformation Processes, Assessed Using Human Adipose Stem Cell Cultures

**DOI:** 10.3390/ijms22137092

**Published:** 2021-06-30

**Authors:** Maria Rosa Iaquinta, Elena Torreggiani, Chiara Mazziotta, Andrea Ruffini, Simone Sprio, Anna Tampieri, Mauro Tognon, Fernanda Martini, Elisa Mazzoni

**Affiliations:** 1 Department of Medical Sciences, Section of Experimental Medicine, School of Medicine, University of Ferrara, 64b Fossato di Mortara Street, 44121 Ferrara, Italy; mariarosa.iaquinta@unife.it (M.R.I.); elena.torreggiani@unife.it (E.T.); chiara.mazziotta@unife.it (C.M.); elisa.mazzoni@unife.it (E.M.); 2 Institute of Science and Technology for Ceramics, National Research Council, 48018 Faenza, Italy; andrea.ruffini@istec.cnr.it (A.R.); simone.sprio@istec.cnr.it (S.S.); anna.tampieri@istec.cnr.it (A.T.); 3 Laboratory for Technologies of Advanced Therapies (LTTA), University of Ferrara, 44121 Ferrara, Italy

**Keywords:** biomorphic scaffolds, hydroxylapatite, bone regeneration, nanostructure, in vitro osteoinductivity

## Abstract

In this study, the in vitro biocompatibility and osteoinductive ability of a recently developed biomorphic hydroxylapatite ceramic scaffold (B-HA) derived from transformation of wood structures were analyzed using human adipose stem cells (hASCs). Cell viability and metabolic activity were evaluated in hASCs, parental cells and in recombinant genetically engineered hASC-eGFP cells expressing the green fluorescence protein. B-HA osteoinductivity properties, such as differentially expressed genes (DEG) involved in the skeletal development pathway, osteocalcin (OCN) protein expression and mineral matrix deposition in hASCs, were evaluated. In vitro induction of osteoblastic genes, such as Alkaline phosphatase (*ALPL*), Bone gamma-carboxyglutamate (gla) protein (*BGLAP*), SMAD family member 3 (*SMAD3*), Sp7 transcription factor (*SP7*) and Transforming growth factor, beta 3 (*TGFB3*) and Tumor necrosis factor (ligand) superfamily, member 11 (*TNFSF11*)/Receptor activator of NF-κB (RANK) ligand (*RANKL*), involved in osteoclast differentiation, was undertaken in cells grown on B-HA. Chondrogenic transcription factor SRY (sex determining region Y)-box 9 (*SOX9*), tested up-regulated in hASCs grown on the B-HA scaffold. Gene expression enhancement in the skeletal development pathway was detected in hASCs using B-HA compared to sintered hydroxylapatite (S-HA). OCN protein expression and calcium deposition were increased in hASCs grown on B-HA in comparison with the control. This study demonstrates the biocompatibility of the novel biomorphic B-HA scaffold and its potential use in osteogenic differentiation for hASCs. Our data highlight the relevance of B-HA for bone regeneration purposes.

## 1. Introduction

In recent times, the steady rise in musculoskeletal disease, resulting from increases in life expectancy and lifestyle changes, has become a serious threat for the ever-increasing population and national health systems. Approximately 20 million patients/year have been reported as suffering from loss of bone tissue due to trauma or diseases [1]. New knowledge about bone repair mechanism is essential to address the important steps required in translational and precise medicine to improve patients healing. Self-repair is known to be challenging when there are massive bone defects due to traumatic injury, tumor resection or congenital disease [2]. Autologous cancellous bone grafting is considered the gold standard for regeneration when dealing with bone defects [3], despite several limitations and drawbacks being frequently encountered, including low bone availability and surgery with its consequent pain and risk of infection [4]. As such, there is growing demand for synthetic bone scaffolds, which are capable of imitating the extracellular matrix and provide an appropriate microenvironment for bone growth by supporting and accelerating cell migration, while facilitating osteogenic differentiation and the regenerative cascade, overall [5,6,7].

Nanotechnology is an innovative discipline that aims to develop/improve new devices with advanced smart performance, thanks to the specific features of certain nanomaterials. Calcium phosphates (CaPs), particularly hydroxylapatite (HA), are currently considered gold-standard materials since their composition mimics the mineral bone phase, thus exhibiting excellent biocompatibility, as well as being able to induce new bone adhesion and excellent bone-integration, particularly when developed as porous implants [8,9,10]. However, despite the large number of studies and medical devices available using HA, regeneration in the case of critical-sized bone defects is still a serious concern. This is due to a lack of bioactivity relating to the classic ceramic-making process by which CaP scaffolds are obtained (i.e., powder compaction/3D forming), particularly the high temperature sintering required to give the scaffold adequate mechanical properties [11]. Indeed, this process causes crystal growth and stabilization in the ceramic phase thus, strongly reducing its ability to be resorbed and exchange bioactive chemical signals with cells, particularly Ca, P and other bioactive ions that drive bone and vascular regeneration.

Problems relating to reduced HA bioactivity upon sintering are being increasingly discussed in the literature. Indeed, various studies are dedicated to the development of self-assembling or self-consolidating ceramic-based scaffolds with adequate mechanical properties, which at the same time maintain the nanostructure [12,13]. In this respect, a sinter-free transformation process has recently been developed, showing a new route to obtain biomorphic ion-doped CaP scaffolds. The process was developed over various steps, during which a wooden structure was subject to pyrolysis and controlled phase transformation through a sequence of heterogeneous gas-solid reactions, culminating in a hydrothermal process conducted at about 200 °C. The final product of this process is a scaffold characterized by a biomimetic composition, multi-scale porosity and a hierarchically-organized structure with lamellar nanosize grains, which is notably different from the standard microstructure found in sintered ceramics, especially due to its unusually damage-tolerant mechanical characteristics [7,14]. 

Previous in vivo studies, carried out on skeletally mature adult New Zealand White disease-free rabbits, have highlighted the osteogenic and osteoconductive character of biomorphic hydroxylapatite scaffolds inherited from the original wood template structure and show extensive bone formation and penetration inside the channel-like pores of the scaffold [15]. Indeed, a more recent study has reported the in vivo osteoinductive ability shown by biomorphic HA scaffold, which was attested by the formation of mature bone tissue in ectopic sites 12 weeks after subcutaneous implantation in rabbits [7]. In vivo data are a clear indication of the relevance of biomimetic physical/chemical, morphological and mechanical features of biomorphic hydroxylapatite scaffold, which stimulates bone tissue regeneration. However, genes and cellular signaling pathways, which drive biomorphic HA osteoinductivity, are not known. The aim of our investigation is address to analyze molecular/cellular pathways and osteogenetic genes induced in human adipose derived stem cells (hASCs) grown on biomorphic scaffold (B-HA).

Special attention is dedicated to mechanisms of epigenetic control operating at the SMAD family member 3 (*SMAD3*) and Sp7 transcription factor (*Sp7*) coding for the two principal master regulators of the osteogenic lineage during mesenchymal stem cell commitment. For example, *SMAD3* has been shown to bind to the osteopontin promoter as a sequence specific activator [16,17]. 

The aim of this study is to analyze the biocompatibility and the ability of B-HA to promote the expression of specific genes, which are relevant to the osteogenic pathway, compared to a commercial scaffold made of a sintered HA (S-HA) with similar porosity levels. In order to detect epigenetic modifications induced by the biomorphic material in hASCs, the analysis of gene expression involved in the osteogenic pathway was carried out. As both scaffolds are made of HA, this study will assess the in vitro osteoinductive ability of a scaffold, as induced by a scaffold endowed with nanostructured hierarchical porous architecture, compared to the typical macroporous cellular structure of a sintered ceramic device, typically showing rounded micron-sized grains, based on a high-temperature sintering process.

## 2. Results

### 2.1. Microstructural Analysis

**Figure 1:**
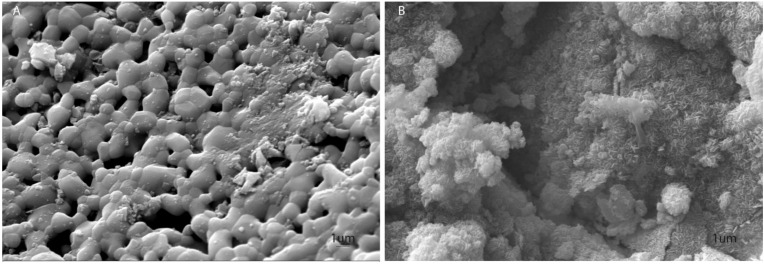
Scanning electron microscopy (SEM) analysis of S-HA and B-HA. (**A**) SEM S-HA images show its porous structure, Scale bar: 1 μm, × 11,57 K. (**B**) B-HA structure is characterized by nano-metric particles forming thin lamellae with a microstructure composed of nano-size building blocks and multi-scale porosity, Scale bar: 1 μm, × 10,210 K.

Sintered HA (S-HA) shows a typical sintered hydroxylapatite microstructure, with coalesced rounded ~1–2 µm sized grains and diffuse intergranular porosity (Figure 1A). Conversely, biomorphic scaffold (B-HA) shows lamellar, closely interconnected hydroxylapatite nanocrystals (~200 × 20 nm) with a hexagonal shape. The absence of any high temperature sintering process prevented grain growth in B-HA, thus resulting in a higher specific surface area (i.e., B-HA: 12.5 m^2^/g; S-HA: 4.8 m^2^/g), without evidence of intergranular boundary layers in strong contrast to the typical microstructure of sintered ceramic bodies (Figure 1B). Both scaffolds showed a porosity extent of ~60 vol.%.

### 2.2. Biocompatibility Analysis of Biomorphic HA Scaffold Employing hASCs

**Figure 2:**
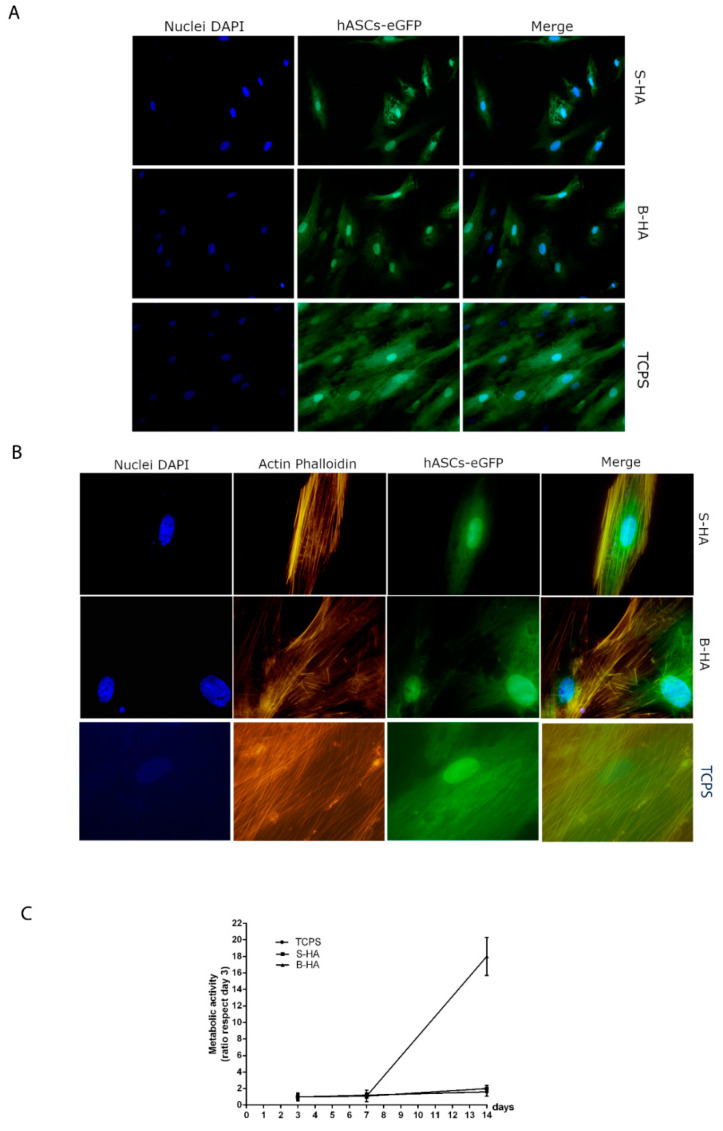
Stem cell viability and cytoskeleton architecture. (**A**) Recombinant genetically engineered human adipose stem cells (hASC-eGFP) grown on biomorphic HA (B-HA), sintered HA (S-HA) and tissue culture polystyrene (TCPS) at day 14 are shown at magnification 20×. Cell nuclei were stained with 0.5 mg/mL DAPI. (**B**) Cytoskeleton analysis by Phalloidin TRITC staining was carried out in hASCs-eGFP grown on biomaterials B-HA and S-HA and TCPS (magnification 40×). Actin filaments show no structural alteration, confirming the compatibility of the assayed biomaterials, at day 14. Cell nuclei were stained with 0.5 mg/mL DAPI. (**C**) human adipose stem cells (hASCs) metabolic activity was evaluated by colorimetric intensity at day 3, 7 and 14 of co-culture on B-HA, S-HA and TCPS. 3D-Biomorphic biomaterials showed the highest value in cell viability between day 7 and day 14 (*p* < 0.05). Statistically differences are significant for B-HA, S-HA and TCPS at day 7 and day 14 (*p* < 0.05).

In vitro biocompatibility analyses, i.e., viability, proliferation and cytoskeleton organization, assayed in human adipose stem cells (hASCs) cultured on biomaterials were evaluated at day 14. The biomorphic HA (B-HA) and sintered HA (S-HA) biomaterials demonstrated biocompatibility in terms of cell growth and proliferation. In this case, recombinant genetically engineered human adipose stem cells (hASC-eGFP) grown on biomaterials revealed a normal cell morphology. Indeed, hASC-eGFP cell morphology was no different from parental hASCs (Figure 2A). Actin fibers of the cytoskeleton appeared to be well organized, whereas its integrity remains uninfluenced by scaffolds (Figure 2B). The Alamar blue assay showed an increased scaffold metabolic activity during the analysis in hASCs grown on B-HA and S-HA scaffold. The metabolic activity measured by Alamar blue assay demonstrated that B-HA and S-HA biomaterials did not elicit cytotoxic effects, although different cellular growth kinetics, which are statistically significant at day 14 compared to day 7 (*p* < 0.05), were induced. Moreover, the B-HA scaffold had a substantial overall effect on cell viability compared to S-HA and TCPS (*p* < 0.05) (Figure 2C).

### 2.3. Matrix Mineralization and Osteocalcin Expression Protein in hASCs 

**Figure 3:**
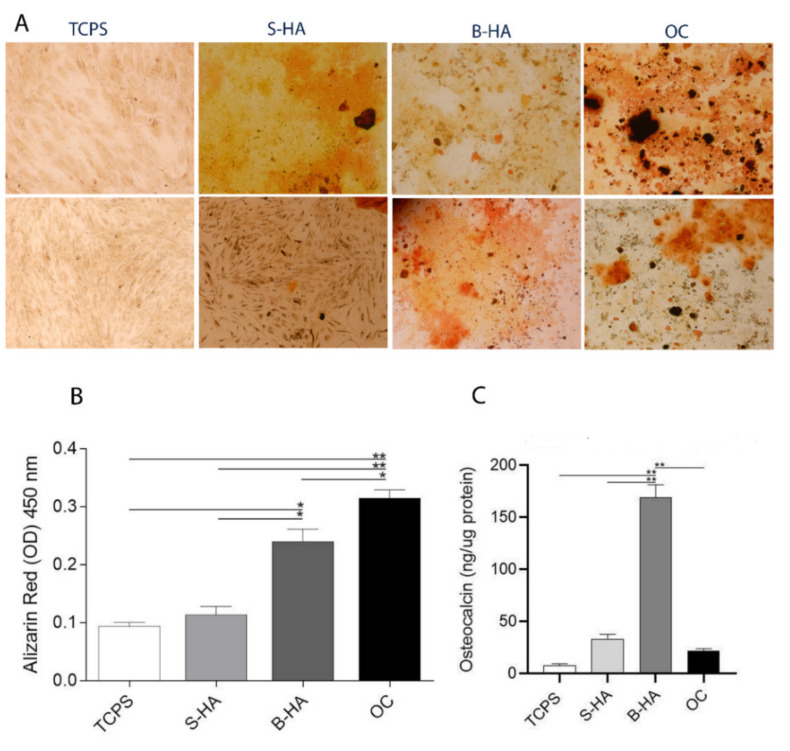
Osteogenic markers in human adipose stem cells (hASCs) cultured on biomorphic HA (B-HA), sintered HA (S-HA) biomaterials. (**A**) Alizarin Red staining is shown in this panel, in experimental conditions tested (10× magnification upper figures, 4x magnification lower figures). (**B**) The matrix mineralization was evaluated by Alizarin red staining, whereas its quantification was carried out spectrophotometrically. * *p* < 0.05; ** *p* < 0.01 (ANOVA test). Matrix mineralization data was reported as optical density. (**C**) The osteocalcin (OCN) protein levels were quantified by ELISAs. OCN protein was reported as ng of Osteocalcin/μg of total protein. * *p* < 0.05, ** *p* < 0.01.

To test the osteogenic differentiation ability of the biomorphic material B-HA, stem cells were grown on scaffolds for two weeks. Mineral matrix deposition was evaluated by staining the cells with Alizarin Red at day 14 in hASCs cultured on B-HA and S-HA biomaterials, in osteogenic condition (OC) and in plastic vessels (TCPS) (Figure 3A). Alizarin Red S staining showed that stem cells formed a small number of calcified nodules (Figure 3 A, B). When co-culture processing was performed for a further two weeks, a large number of calcified nodules were observed and quantified (Figure 3 A, B). The B-HA scaffold favored matrix mineralization better than TCPS, the control group (** *p* < 0.01). Calcium deposits quantified in hASCs grown on the B-HA material were higher compared to stem cells grown on the S-HA biomaterial (** *p* < 0.01). Cells grown in OC showed a significant increase in calcium deposits compared to S-HA, TCPS (** *p* < 0.01) and B-HA (* *p* < 0.05), (Figure 3B). The B-HA scaffold favored matrix mineralization better than TCPS, also at day 21 (Supplementary Figure S1). 

Osteocalcin (OCN) is the most important non-collagenous protein involved in bone matrix organization and deposition. ELISAs show a statistically significant increasing level of OCN protein expression in cells grown on B-HA biomaterial, compared to the other experimental groups, represented by S-HA and OC (* *p* < 0.05) and TCPS (** *p* < 0.01) at day 14. These improved cellular responses for hASCs grown on B-HA material demonstrate the inductive effect exerted by B-HA compared to S-HA scaffold (Figure 3C).

### 2.4. Osteogenic Gene Expression in hASCs

**Figure 4:**
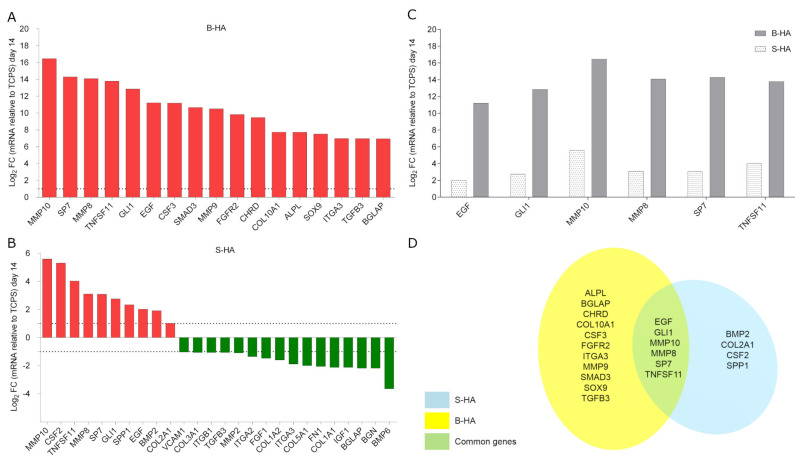
RT^2^ PCR Array analyses of osteogenic genes. Gene expression was evaluated in human adipose stem cells (hASCs) grown on (**A**) biomorphic HA (B-HA) and (**B**) sintered HA (S-HA), compared to tissue culture polystyrene (TCPS) at day 14. (**A**) In hASC cultures, mRNAs from 17 genes in the osteogenic pathway, i.e., *ALPL, BGLAP, CHRD, COL10A1, CSF3, EGF, FGFR2, GLI1, ITGA3, MMP10, MMP8, MMP9, SMAD3, SOX9, SP7, TGFB3* and *TNFSF11* were up-regulated (red). (**B**) In hASC cultures, mRNAs of 10 genes in the osteogenic pathway, i.e., *BMP2, COL2A1, CSF2, EGF, GLI1, MMP10, MMP8, SP7, SPP1, TNFSF11* were up-regulated (red), while 16 tested genes resulted down-regulated (green): *BGLAP, BGN, BMP6, COL1A1, COL1A2, COL3A1, COL5A1, FGF1, FN1, IGF1, ITGA2, ITGA3, ITGB1, MMP2, TGFB3* and *VCAM1*. (**C**) Fold change (FC) of common up-regulated gene *EGF, GLI1, MMP10, MMP8, SP7, TNFSF11*. (**D**) Genes modulated by B-HA and S-HA in hASC cultures at day 14 are represented by Venn diagram. The common genes up-regulated from both biomaterials are indicated.

The expression profile of human osteogenic genes was evaluated by RT-PCR Array technology. To this end, hASCs were grown on both B-HA and S-HA scaffolds for 14 days. Quantitative RT-PCR Array results were compared to the control group, using hASCs grown on TCPS. In hASCs grown on B-HA, 17 differentially expressed genes (DEGs) were detected (Figure 4A). Among the DEGs, ossification and osteoblast differentiation-related genes, including the Alkaline phosphatase (*ALPL*), Chordin (*CHRD*), Fibroblast growth factor receptor 2 (*FGFR2*) and SMAD family member 3 (*SMAD3*) resulted as up-regulated in hASCs grown on B-HA compared to the control group (TCPS). Transcription factor to cartilage condensation SRY (sex determining region Y)-box 9 (*SOX9*) tested up-regulated, too. HASC adhesion analysis and extracellular matrix gene expression reveal that collagen, type X, alpha 1 (*COL10A1*) and Matrix metallopeptidase 9 (*MMP*9), together with the growth factor colony-stimulating factor 3 (granulocyte) (*CSF3*), were up-regulated on B-HA scaffold. 

A total of 26 DEGs were identified in hASCs grown on sintered hydroxylapatite S-HA biomaterial (Figure 4B). DEGs including 10 up-regulated genes (red) and 16 down-regulated genes (green) were identified in hASCs grown on S-HA. Among these genes were accounted Bone morphogenetic protein 2 (*BMP2*), Collagen, type II, alpha 1 (*COL2A1*) and Secreted phosphoprotein 1 (*SPP1*), which play important roles in ossification, together with the growth factor colony-stimulating factor 2 (*CSF2*), were found to be up-regulated at day 14. Genes encoding for cell-cell adhesion molecules, such as Integrin, alpha 2 (*ITGA2*), Integrin beta 1 (*ITGB1*) and Vascular cell adhesion molecule 1 (*VCAM1*) were down-regulated at day 14. Genes that codify extracellular matrix (ECM) proteins, such as Collagen type I, alpha 1 (*COL1A1*), Collagen type I, alpha 2 (*COL1A2*), Collagen type III, alpha 1 (*COL3A1*), Collagen type V, alpha 1 (*COL5A1*), Biglycan (*BGN*), Fibronectin 1 (*FN1*) and Matrix metallopeptidase 2 (*MMP2*) were down-regulated. Growth factors, such as fibroblast growth factor 1 (*FGF1*) and insulin-like growth factor-1 (*IGF1*), together with Bone morphogenetic protein 6 (*BMP6*) were down-regulated. It appears that both biomaterials stimulate the over-expression of specific osteoblastic genes, such as Sp7 transcription factor (*SP7*) and GLI family zinc finger 1 (*GLI1*) with different fold change values (Table 1, Figure 4C,D). DEGs modulated by the two scaffolds also include growth factors such as Epidermal growth factor (*EGF*) and ECM molecules, including Matrix metallopeptidase 8 (*MMP8*) and Matrix metallopeptidase 10 (*MMP10*), and factors implicated in osteoclastic differentiation, such as Tumor necrosis factor (ligand) superfamily, member 11 (*TNFSF11*). B-HA induced the up-regulation of these common genes, i.e., *SP7, GLI1, MMP8-10, EGF* and *TNFSF11*, with a higher fold change compared to S-HA material (*p* < 0.05) (Table 1, Figure 4C). Bone gamma-carboxyglutamate (gla) protein (*BGLAP*), Integrin, alpha 3 (*ITGA3*) and Transforming growth factor beta 3 (*TGFB3*) tested differentially regulated, i.e., up- and down-regulated in hASCs grown on B-HA and S-HA, respectively.

## 3. Discussion

In this investigation, a recently developed biomorphic nanostructured hydroxylapatite (B-HA) was analyzed in vitro by differential gene expression, compared to a sintered hydroxylapatite (S-HA), exhibiting a structure made of micron-size rounded grains, as typical of hydroxylapatite consolidated by the sintering process. 

Viability analysis studies on metabolic activity and surface studies conducted with hASCs, when taken together suggest that both scaffolds meet the requirements for in vitro biocompatibility, offering surface properties with good microenvironments for human adipose stem cell (hASC) adhesion and proliferation when in direct contact with these materials. The distribution of actin filaments was similar in hASCs grown on B-HA and plastic vessel (TCPS). In the examined hASCs grown on the biomorphic B-HA scaffold, the actin filaments were distributed uniformly and occupied most of the cell cytoplasm at day 14. On the other hand, hASCs grown on S-HA showed cellular viability, metabolic activity and well-organized cytoskeleton confirming its biocompatibility.

The in vitro osteoinductive ability of both the B-HA and S-HA scaffolds was assessed by an analysis of calcium deposits and Osteocalcin (OCN) protein expression. hASCs grown on B-HA and S-HA at day 14 accumulated calcium in the ECM, thus demonstrating osteoinductive properties in both scaffolds. However, calcium deposits in hASCs grown on B-HA were more abundant in comparison to cells grown on the S-HA scaffold. 

Furthermore, ELISA data show a statistically significant increase of OCN with B-HA compared to controls, represented by S-HA and TCPS, at day 14, in agreement with the up-modulation of Bone gamma-carboxyglutamate (gla) protein (*BGLAP*) gene expression. Indeed, in hASCs grown on B-HA up-regulation of *BGLAP* expression at mRNA level was detected. On the other hand, this gene tested down-expressed in hASCs grown on S-HA. OCN, together with osteopontin (OPN), are known to be major non-collagenous proteins (NCPs) that play key roles in both the biological and mechanical functions of bone [18]. 

Indeed, OCN is produced during bone formation, late in the mineralization process, as it is involved in organizing ECM, coordinating cell–matrix and mineral–matrix interactions, particularly controlling either directly and/or indirectly bone mass, mineral size and orientation [18]. 

These improved osteogenic markers, expressed by hASCs grown on the B-HA scaffold, demonstrate a higher osteoinductive ability for B-HA compared to S-HA scaffold.

The molecular mechanisms, activated by hydroxylapatite (HA) to influence the behavior of osteoblasts inducing proliferation and bone formation, are poorly understood. For this reason, we performed RT^2^ Profiler PCR array-Human Osteogenesis in order to analyze the expression of genes involved in the osteogenic pathway. In this study, S-HA biomaterial influences the differentiation of hASCs by up-regulating osteogenic genes.

S-HA at day 14 induced the up-regulation of ten genes including *BMP2, COL2A1, SPP1* and *SP7,* which play important roles in the ossification process. In agreement, previous studies have reported that sintered hydroxylapatite induced the expression of osteogenic genes, including *SPP1*, in hASCs [19], while high proliferation and focal adhesion kinase activation were observed in human bone marrow mesenchymal stem cells (BM-MSCs) [20]. 

In this study, the novel biomimetic scaffold modulated the ossification differentiation genes including the chondrogenic transcription factor and genes involved in the osteoclast pathway, at day 14 of co-culture. Among the DEGs, gene expression analysis performed using RT-PCR Array showed significant early up-regulation of the osteogenic markers *ALPL, BGLAP, CHRD, FGFR2, SMAD3, TGFB3* in hASCs grown on B-HA at day 14. Recent studies have indicated that *ALPL* is an essential factor for the mineralization of osteoblastic cells [21]. In agreement with this result, our data show the up-regulation of *ALPL* gene expression in hASCs grown on B-HA scaffold. Animal models have indicated that the BMP-antagonist chordin (*CHRD*) and Noggin promote inductive and trophic activities in rostral organizing centers during early development of the mammalian head [22]. In our study, *SMAD3* and *TGFB3* were up-regulated in hASCs grown on B-HA scaffolds compared to the control group represented by TCPS. *SMAD3* is an intracellular molecule that transmit signals from plasma membrane receptors to the nucleus. *SMAD3* operates down-stream of growth factors, such as transforming growth factor-β (*TGFB*) [23]. It is worth noting that *TGFB3* tested up-regulated in hASCs grown on B-HA, whereas it was down-regulated in cells grown on the S-HA scaffold. 

*TGFB3* loaded on a 3D scaffold enhanced the chondrogenic differentiation of hASCs during 28-days of culture [24]. Interestingly, it has been reported that *TGFB3* promotes osteogenic differentiation [25] and it is used for cartilage repair, tissue regeneration and wound healing in vivo [24,26,27]. *TGFB3* promotes the proliferation and early differentiation of mesenchymal stem cells (MSCs) into osteoblasts, chondrocytes, adipocytes and tendon cells [28]. *TGFB3* is also involved in the recruitment of endogenous MSCs to initiate bone tissue regeneration [29]. Furthermore, it stimulates endochondral ossification [30] and completes bone remodeling [31].

In this investigation, we show that the B-HA modulated *EGF, GLI1, MMP8-10, SP7* and *TNFSF11* gene expression with higher fold change values compared to S-HA material. The intracellular stimulation of EGF gene expression is much more active with B-HA scaffolds than S-HA (11,21 vs. 2,01 Log_2_ Fold change). *EGF* has been reported as playing an enhancer role on osteogenic differentiation since it increases extracellular matrix mineralization [32]. 

Array RT-PCR reveals that the *GLI1* gene expression is up-regulated by B-HA compared to S-HA. It has been reported that *GLI1*, in addition to *GLI2* and *GLI3*, is involved in the signaling-mediated specification of the osteoblast lineage. *GLI1* induces an early stage of osteoblast differentiation, at least to some extent, in a *Runx2*-independent manner [33]. Chi et al., reported that the Hedgehog signaling pathway can promote osteogenic differentiation in bone marrow mesenchymal stem cells (BM-MSCs) via the activation of key molecules Smoothened (Smo) and *GLI1* Family Zinc Finger 1 [34]. 

B-HA modulated matrix metalloproteinases (*MMP*) expression genes more actively than S-HA in hASCs. *MMPs* are members of a family of zinc-dependent proteinases, which are able to cleave many non-ECM and ECM components, such as collagens and proteoglycans. They have a role in normal development and tissue damage in various pathophysiological conditions involving wound healing and tissue remodeling [35]. *MMP8* has been reported as being expressed by osteoblastic progenitors, differentiated osteoblasts, osteocytes and chondrocytes [36]. In our in vitro study, *ITGA3* was up- and down-regulated in hASCs grown on B-HA and S-HA, respectively. In this context, it is important to recall that at the cellular level, the integrins are significant mechano-transducers involved in both matrix deposition and organization. 

*SP7*, an important transcriptional factor that controls the proliferation and differentiation of MSCs in mature bone cells, tested as over-expressed in hASCs grown on both S-HA and B-HA scaffolds. In this context, it is worth recalling that *SP7* controls the expression of proteins involved in terminal osteoblast differentiation [37]. 

Osteoclast differentiation, mediated by *TNFSF11/RANKL* gene, was greatly up-regulated (13,78 Log_2_ FC) by B-HA compared to S-HA (4,02 Log_2_ FC). *RANKL* is a homotrimeric transmembrane protein secreted by osteoblasts as well as immune and tumor cells, which stimulate the differentiation of osteoclasts in the bone and the release immature progenitor cells into the circulation. Bone is a dynamic tissue that is continuously being remodeled by the coordinated actions of different bone cell populations, which include bone resorption by osteoclasts and bone formation by osteoblasts, whereas osteocytes act as mechanic-sensors that orchestrate osteoclast and osteoblast behavior.

The high expression of various genes implicated in skeletal development, such as *EGF, GLI1, MMP8, SP7* and *TNSF11* induced by B-HA compared to S-HA can be ascribed to the very different physicochemical features of the two scaffolds. Indeed, despite having the same composition, i.e., hydroxylapatite, B-HA is characterized by nanosize lamellar particles resembling the structure of the mineral phase in natural bone tissue. Due to such a fine nanostructure, in comparison with the micron-size particles making up the sintered S-HA structure, B-HA shows a much higher specific surface area and can also exchange Ca and P ions which are relevant as chemical signals for stem cells favoring osteogenic gene expression, as previously shown with a Mg, Sr-doped biomorphic HA-based scaffold [7]. Furthermore, in comparison with S-HA, characterized by wide open, but random and non-aligned porosity, B-HA shows a hierarchically organized structure at increasing size scales, following the complex structure of the rattan wood used as a template and closely mimicking the osteon structure of bone. Therefore, hASCs cultured on the B-HA scaffold could also receive topological information from its 3D architecture, which closely reproduces the physiological bone environment from the nano to the macroscale and is relevant in instructing cells on new bone formation and organization [38]. 

Overall, our data show that six important genes (*EGF, GLI1, MMP8-10, SP7* and *TNSF11*) were up–regulated in B-HA with higher fold change values compared to S-HA material. In addition, *BGLAP, ITGA3* and *TGFB3* tested up- regulated in hASCs grown on B-HA and down regulated in S-HA. Some genes involved in ostegenic and chondrogenic differentiation (e.g., *ALP, SOX9, SMAD3, COL10A1*) are expressed only in hASCs grown on B-HA, compared to S-HA. This is a good marker of the potentially effective, sustained osteogenic activity of B-HA. This result indicates that B-HA is a promising scaffold for bone regeneration. Indeed, B-HA has a bone-like composition and nanostructured 3D architecture, whereas it does not need growth factors or osteoinductive additives.

Our new data indicate that B-HA improves the osteogenic, osteoclastic and chondrogenic genes expression promoting bone regrowth. These results could be ascribed to the higher surface activity of the B-HA scaffold compared to S-HA.

However, as the osteogenesis process is the result of a very complex sequence of biochemical reactions induced by both the chemistry and the structure of the scaffold, a more detailed investigation with different time points is required in order to have a global understanding of the molecular events related to osteogenesis, as induced by specific chemico-physical features of the scaffold [14,15].

The enhanced osteoinductive ability of B-HA compared to a S-HA scaffold confirms that the use of biomimetic scaffolds can support metabolic processes yielding tissue regeneration without the aid of additional growth factors, bioactive molecules and cells. These results could have a significant impact on translational processes, because it would be possible to avoid regulatory complexes, linked to the use of osteogenic molecules and human stem cells. This scaffold may significantly reduce the time required for the translation “from the bench to the patient”, whereas decreasing subsequent healthcare costs.

## 4. Materials and Methods

### 4.1. Biomaterials

The biomorphic scaffold (B-HA) was obtained following the method described elsewhere [7], while being slightly modified to obtain a pure hydroxylapatite (HA) scaffold with no further ion doping. Briefly, cylindrical rattan wood pieces (*Calamus manna*) were pyrolyzed at 1000 °C in an N_2_ atmosphere, generating a pure carbon template. Then, the carbon template was transformed into a biomorphic hydroxylapatite scaffold by a sequence of gas-solid reactions in a controlled atmosphere at supercritical conditions, which concluded with a hydrothermal process carried out at 220 °C. 

The biocompatibility and osteoinductivity properties of B-HA were assessed compared to a commercial sintered hydroxylapatite scaffold (Engipore^®^; Finceramica Faenza, Faenza, Italy; hereinafter coded as S-HA) [39,40]. Before cell loading, each sample (diameter 8 mm, height 4 mm), was sterilized using 25 kGy γ-ray radiation, placed in a 24-well plate (one per well) and pre-soaked in culture medium for 72 h at 37 °C.

### 4.2. Microstructural Analysis

B-HA and S-HA scaffolds were analyzed using scanning electron microscopy (SEM). Samples were washed with saline, fixed by 2.5% glutaraldehyde and with a 1% osmium solution in phosphate buffer. The specimens were coated with colloidal gold and analyzed using scanning electron microscopy (SEM, model Stereoscan S-360, Cambridge UK) [41]. 

The open and total porosity of the studied ceramics was measured using Archimedes’ method and geometrical weight-volume evaluation, respectively. The specific surface area (SSA) of the scaffold was measured using the nitrogen adsorption method, following the Brunauer–Emmett–Teller (BET) model (Sorpty 1750, Carlo Erba, Milan, Italy).

### 4.3. Cell Culture and Scaffold Seeding

The biocompatibility and osteoinductivity of the B-HA scaffold, compared to S-HA, were evaluated using human adipose derived stem cell (hASC) cultures at day 14. The hASCs used in this study were purchased (PT-5006, Lonza Milan, Italy) as cryopreserved frozen cells during the first passage. The company certified that hASCs are positive for surface markers CD13, CD29, CD44, CD73, CD90, CD105 and CD166, while being negative for other markers, such as CD14, CD31 and CD45. Cells were expanded in αMEM (Lonza, Milan, Italy) supplemented with 20% fetal bovine serum (FBS), antibiotics and incubated at 37 °C with 5% CO_2_ in a humidified atmosphere. Primary hASC cultures were grown (i) on B-HA; (ii) on S-HA; (iii) in osteogenic condition (OC). HASC cultures grown on B-HA and S-HA biomaterials were maintained in a-minimum essential medium (α-MEM) (Lonza, Milan, Italy) supplemented with 20% FBS and 2% antibiotics (Pen/Strep 10,000 U/mL). Control cultures were hASCs grown in tissue culture polystyrene (TCPS) vessels, and maintained with basal medium, described above. OC was obtained using differentiation Bullekit osteogenic medium (Lonza, Milan, Italy), containing osteogenic basal medium (Lonza, Milan, Italy) and osteogenic SigleQuotes, which includes dexamethasone, ascorbate, mesenchymal cell growth supplement, L-glutamine, β-glycerophosphate (Lonza, Milan, Italy) [41,42]. The S-HA and B-HA scaffolds were placed separately in 24-well plates (Ø, 10 mm), filled with 200 µL of cell suspension containing 10^4^ hASCs for each sample and incubated for 2 h. Cell suspension was shaken every 15 min to maximize cell-scaffold interaction. Plates were incubated at 37 °C in a humidified atmosphere until the time of analysis [39]. Cells were cultured at 37 °C in a humidified atmosphere with 5% CO_2_, whereas they were re-fed with fresh medium every three days until the time of analysis. Biocompatibility analyses were performed in triplicate employing three different hASC samples.

### 4.4. Cell Morphology

HASCs (10^4^ cells) were seeded onto the B-HA and S-HA scaffolds in order to evaluate the influence of biomaterials on viability, cytoskeleton organization and proliferation, as reported below. HASCs employed in the three experimental groups, i.e., biomaterial B-HA, biomaterial S-HA and control TCPS, were assayed at day 14. In order to analyze the biocompatibility of both B-HA and S-HA scaffolds, the direct morphology of recombinant genetically engineered cells hASCs-eGFP grown on the two biomaterials was evaluated by fluorescence microscopy analysis. To facilitate the observation of hASC cultures grown onto biomaterials, cells were transfected with an adenovirus vector that expresses the enhanced green fluorescence protein (eGFP). Recombinant Ad-eGFP was prepared as previously described [43]. After 48 h, the efficiency of the adenovirus infection was evaluated by measuring the emitted fluorescence by a fluorescence microscope. Cell nuclei were stained with 0.5 mg/mL DAPI and observed at day 14 [41,42].

### 4.5. Cytoskeleton Architecture Evaluation

Cytoskeletal actin filaments of hASCs-eGFP were stained with tetramethyl-rhodamine-isothiocyanate (TRITC) conjugated-Phalloidin (Sigma, Milan, Italy) as previously described at day 14. Cells were washed with PBS 1X and fixed for 10 min at room temperature (RT) using 10% formalin [39,41,44]. Cellular nuclei were stained with 0.5 mg/mL DAPI. Images were obtained using a TE 2000-E fluorescent microscope. Digital images were captured using ACT-1 and ACT-2 software for DXM1200F digital cameras (Nikon Instruments, Sesto Fiorentino, Italy).

### 4.6. Cell Proliferation Assay

The proliferation rate of hASCs grown on scaffold was evaluated using the AlamarBlue assay (ThermoFisher Scientific, Milan, Italy) [39,44]. The assay was performed to assess the cells viability attached and grown on biomaterials and TCPS at day 3, 7 and 14, as previously reported [39,44]. Briefly, cells were incubated with a solution of 5% AlamarBlue in medium for 3 h at 37 °C. Afterwards, the optical density of the supernatants was measured at 570 nm and 620 nm using the spectrophotometer (Thermo Electron Corporation, model Multiskan EX, Helsinki, Finland).

### 4.7. Osteogenic Differentiation and Matrix Mineralization in hASCs

Alizarin Red staining was carried out once calcified nodules were observed at day 14 and 21. Alizarin red staining (Sigma, Milan, Italy) was performed to investigate hASC scaffold-induced mineralization, as described [41]. The medium was removed and cells were fixed with 10% formalin. Plates were rinsed three times with PBS 1X and stained with Alizarin Red (pH 4.2) for 30 min at RT. Excess dye was removed, in case of over-staining, by washing three times with PBS 1X. Cell images were captured using an inverted fluorescence microscope. The mineralized substrates were quantified using a 20% methanol and 10% acetic acid in a water solution (Sigma-Aldrich, Milan, Italy). For the quantification of matrix mineralization, the solution containing an amount of dissolved Alizarin red was read spectrophotometrically (Thermo Electron Corp., model Multiskan EX, Vantaa, Finland) at a wavelength (λ) of 450 nm [41].

More elaborate methods to demonstrate hASC osteogenic differentiation, including the detection and quantification of bone-specific proteins, such as osteocalcin (OCN), were carried out. OCN protein expression was analyzed in hASCs cultured with OC (medium described above) on the S-HA, B-HA scaffolds and TCPS. Protein extraction was performed using Cell extraction Buffer (Thermo Fischer Scientific, Milan, Italy) added to 1 Mm phenylmethylsulfonylfluoride and a protease inhibitor cocktail [41]. Total protein concentration was determined by bicinchoninic acid assay (BCA) according to the manufacturer’s instructions. The OCN protein was quantified using the Human Osteocalcin Instant ELISA (Thermo Fisher Scientific, Milan, Italy) according to the manufacturer’s instructions [41].

### 4.8. Osteogenesis RT^2^PCR Array

RT^2^ Profiler™ PCR Array Human Osteogenesis (Qiagen, Milan, Italy) was carried out to investigate the gene expression of the osteogenic pathway induced by the scaffold in hASCs. Specifically, total RNA was isolated from hASCs grown on (i) B-HA (ii) S-HA and (iii) TCPS (control group), at day 14, using RNeasy Plus Micro Kit (Qiagen, Milan, Italy) according to the manufacturer’s instructions. Total RNA was quantified using a Nanodrop spectrophotometer (NanoDrop Technologies, Wilmington, Delaware) [44]. RT^2^ Profiler™ PCR Array Human Osteogenesis was performed according to the manufacturer’s instructions [41]. Briefly, the RNA sample was purified from the genomic DNA with buffer GE for 5 min at 42 °C. Reverse-transcription mix (10 μL) was added to each tube containing 10 μL genomic DNA elimination mix. The reverse transcription was performed 42 °C for 15 min. RT^2^ SYBR Green Mastermix was added at cDNA in RNase-free water. PCR components mix (25 µL) was added to each well of the RT^2^ PCR Array. RT^2^ Profiler PCR Array was used to analyze the expression of genes for human osteogenesis, cell adhesion molecules and 5 housekeeping genes, at days 14 [41]. Specific primer sets, employed in Real-Time PCR, were used to analyze the expression of 84 genes codifying for proteins involved in osteogenic differentiation, cartilage condensation, ossification, bone metabolism, bone mineralization, binding to Ca^2+^ and homeostasis, extracellular matrix (ECM) protease inhibitors, adhesion molecules, cell-to-cell adhesion, adhesion molecules of the ECM and growth factors. For data analysis, the fold change (FC) of each gene expression was calculated using the 2^−ΔΔCt^ method, whereas housekeeping genes, used as controls, were employed to normalize results and Log_2_ FC; <1 or >1 was considered significant. 

### 4.9. Statistical Analysis

Statistical analyses of data, obtained from experiments performed in triplicate, were carried out using Prism 8.0 (GraphPad Software, La Jolla, California) [41]. One-way ANOVA with Dunnett’s as post-hoc test was used. A value of *p*-value < 0.05 was considered significant. The OCN protein level was analyzed with 1-way ANOVA. 

## 5. Conclusions

In vitro tests carried out with hydroxylapatite (HA) porous scaffolds, obtained using a recently developed biomorphic transformation process, reported enhanced osteoinductive ability, attested by overexpression of various genes involved in the osteogenesis process after 2 weeks, in comparison with a scaffold made of HA with similar porosity, obtained using the classical sintering process. We ascribe these results to the higher surface bioactivity of the biomorphic scaffold (B-HA), promoted by its fine nanostructure, showing lamellar HA nanoparticles as building blocks of the scaffold and resulting in a much higher specific surface area. The creation of a 3D ceramic scaffold retaining nanostructure was possible thanks to the use of a new fabrication process transforming a natural wood into a ceramic scaffold, without using high temperature sintering thus preventing the growth of HA crystals and the loss of bioactivity. The unique features of B-HA are relevant in enhancing the capacity to modulate various osteogenic genes, transcription factors and genes related to osteoclast differentiation in hASCs indicating that the use of scaffolds associating biomimetic composition and nanostructure enabling higher surface activity is a promising route towards the development of new bio-devices with superior performance in the regeneration of bone tissue. 

Our data indicate that the innovative B-HA scaffold, tested herein, provides a good microenvironment for hASC viability and osteoinduction. In vitro induction of osteoblastic genes, such as *ALPL, BGLAP, SMAD3, SP7* and *TGFB3*, and the osteoclast differentiation gene, i.e., *TNFSF11/RANKL*, was detected in cells grown on B-HA. B-HA also modulated *MMPs*, which are able to cleave many non-ECM and ECM components, such as collagens and proteoglycans. *MMPs* have a role in normal development and tissue damage in distinct pathophysiological conditions involving wound healing and tissue remodeling. The over-expression of these osteogenic genes in hASCs allow us to better understand the molecular mechanisms that lead to the formation of new bone observed before in the animal model. Moreover, hASCs appear an excellent in vitro cellular model to assay scaffolds, thus stem cells seem to be a significant model to estimate the performance of implant materials in vitro. Our data highlight the translation potential of the biomorphic HA biomaterial in clinical applications.

## Figures and Tables

**Table 1:** Genes found to be de-regulated in human adipose stem cells (hASCs) grown on biomorphic HA (B-HA) and sintered HA (S-HA) scaffolds at day 14.

Common De-Regulated Genes			
Number	Acronym	Fold-Change (Log_2_ FC)	
		S-HA	B-HA
1	BGLAP	−2.18	+6.94
2	EGF	+2.01	+11.21
3	GLI1	+2.75	+12.85
4	ITGA3	−1.89	+6.97
5	MMP10	+5.59	+16.46
6	MMP8	+3.10	+14.08
7	SP7	+3.08	+14.29
8	TGFB3	−1.06	+6.95
9	TNFSF11	+4.02	+13.78

Bone gamma-carboxyglutamate (gla) protein (*BGLAP*), Epidermal growth factor (*EGF*), GLI family zinc finger 1 (*GLI1*), Integrin alpha 3 (*ITGA3*), Matrix metallopeptidase 10 (*MMP10*), Matrix metallopeptidase 8 (*MMP8*), Sp7 transcription factor (*SP7*), Transforming growth factor, beta 3 (*TGFB3*), Tumor necrosis factor (ligand) superfamily, member 11 (*TNFSF11*).

## Supplementary Materials

The following are available online at https://www.mdpi.com/article/10.3390/ijms22137092/s1, Figure S1. Osteogenic markers in hASCs cultured on B-HA and S-HA biomaterials.
