# Supplementary material for: In Vitro Osteoinductivity Assay of Hydroxylapatite Scaffolds, Obtained with Biomorphic Transformation Processes, Assessed Using Human Adipose Stem Cell Cultures
Source: Int J Mol Sci. 2021 Jun 30;22(13):7092. doi: 10.3390/ijms22137092 (PMC8267654; doi:10.3390/ijms22137092)
Supplement: Supplementary file 1 [file ijms-22-07092-s001.zip › ijms-1167682-supplementary.pdf]

Supplementary File 1.

In vitro osteoinductivity assay of hydroxylapatite scaffolds, obtained with biomorphic transformation processes, assessed using human adipose stem cells.

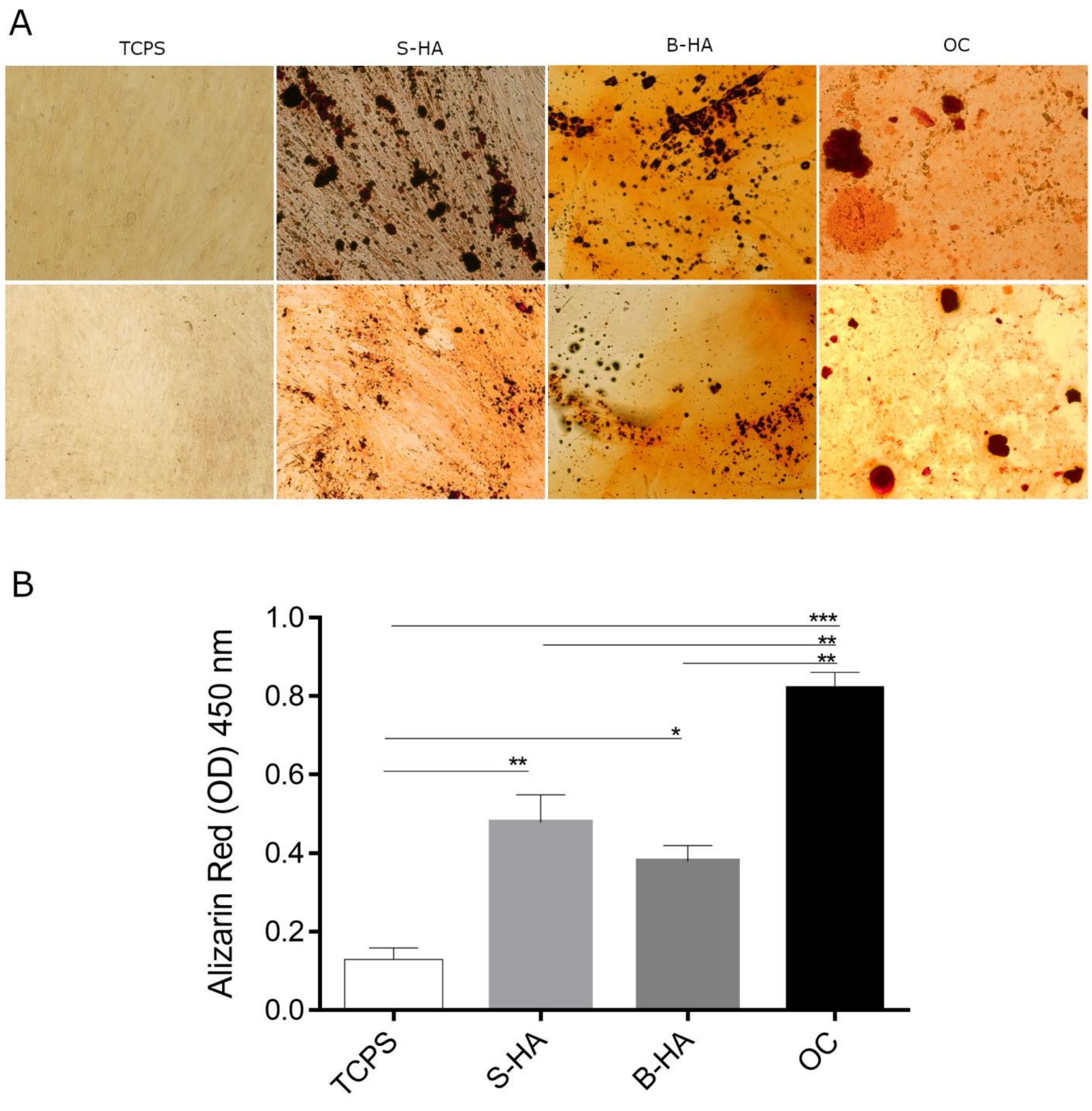

Figure S1. Osteogenic markers in hASCs cultured on B-HA and S-HA biomaterials.

(A) Alizarin red staining is shown in the panel, in experimental conditions tested (10x magnification upper figures, 4x magnification lower figures).

(B) The matrix mineralization was evaluated by Alizarin red staining at day 21, whereas its quantification was carried out spectrophotometrically. Matrix mineralization data was reported as optical density. *$p<0.05$; **$p<0.01$; *** $p<0.001$ (ANOVA test was used).
